# An Explanatory Model of Sexual Satisfaction in Adults with a Same-Sex Partner: An Analysis Based on Gender Differences

**DOI:** 10.3390/ijerph17103393

**Published:** 2020-05-13

**Authors:** Cristóbal Calvillo, María del Mar Sánchez-Fuentes, Juan Carlos Sierra

**Affiliations:** 1 Mind, Brain, and Behavior Research Center (CIMCYC), University of Granada, 18011 Granada, Spain; ccalvillo@correo.ugr.es; 2 Faculty of Social and Human Sciences, University of Zaragoza, 44003 Teruel, Spain; marsanchez@unizar.es; 3 Department of Social Sciences, Faculty of Human and Social Sciences, University of La Costa, Barranquilla 080002, Colombia

**Keywords:** sexual health, sexual satisfaction, attachment, men, women, same-sex attracted individuals, explanatory model, IEMSS, ecological theory

## Abstract

This study aimed to develop an explanatory model of sexual satisfaction in same-sex attracted individuals with a partner, based on personal and interpersonal variables. The participants were 410 men (mean age = 29.24, SD = 9.84) and 410 women (mean age = 29, SD = 8.57) who maintained a relationship with another person of the same sex. Internalized homophobia was considered as a personal variable, and as interpersonal variables, the dimensions of attachment (anxiety and avoidance), sexual functioning, dyadic adjustment, relationship satisfaction, the components of the Interpersonal Exchange Model of Sexual Satisfaction, the number of sexual costs and the number of sexual rewards were considered. The degree to which sexual satisfaction was related to these variables was examined separately, for both men and women, through multiple linear regression models within the framework of structural equation models. The results indicated that sexual satisfaction is associated in a negative sense with internalized homophobia, the number of sexual costs, anxiety, and avoidance, and in a positive sense with the remaining variables. Relational variables were more relevant in the explanation of sexual satisfaction. The clinical implications are discussed.

## 1. Introduction

Sexual health is not merely the absence of disease, but a state of physical, mental, and social well-being concerning an individual’s sexuality [1]. On the other hand, sexual satisfaction is an indicator of good sexual health, which has in turn been associated with people’s general well-being [2], as well as with different personal, interpersonal, and sociocultural variables [3,4]. In the context of couple relationships, Lawrance and Byers define sexual satisfaction as “an affective response arising from one’s subjective evaluation of the positive and negative dimensions associated with one’s sexual relationship” [5] (p. 268). For Pascoal et al., it constitutes an “emotional experience of frequent mutual sexual pleasure” [6] (p. 6). Both definitions relate satisfaction with sexual pleasure, understood as “the physical and psychological satisfaction and enjoyment derived from solitary or shared erotic experiences, including thoughts, dreams, and autoeroticism” [7]. 

Most studies focused on sexual satisfaction have focused their interest on the Caucasian/Anglo-Saxon heterosexual population [8,9,10], and research among the same-sex attracted population is much scarcer [8,11]. This gap impedes a thorough comprehension of sexual health, sexual well-being, and relationships among sexual minorities, and curbs the development of interventions to support these people as they establish healthy relationships in the face of social stigma [12]. In a recent review by Calvillo et al. [3], the authors identified factors associated with sexual satisfaction in same-sex attracted individuals using the ecological model of human development [13] as a reference to organize sexual satisfaction into four interconnected systems: Personal, interpersonal, social, and ideological-cultural. This classification has been used in other studies on sexual satisfaction or subjective orgasmic experience [4,14,15,16,17] due to its adequacy to determine the interaction between different variables associated with each other and with individuals themselves.

In this organizational system, based on the Ecological theory [3,16,17], the first system concerns personal variables, those most closely related to the person (e.g., sociodemographic characteristics, attitudes, or emotions); the second system is associated with interpersonal variables, which are manifested within the interaction with people closest to the individual (e.g., the partner); the third system is concerned with social variables, referred to as distal factors that can indirectly influence the person (e.g., social or family support networks); the last system is comprised of the ideological-cultural variables associated with social and cultural principles, which would exert a distant influence on the individual [18].

Personal and interpersonal variables have been shown to outweigh the social and ideological-cultural variables in the explanation of sexual satisfaction [3,4]. The present study considered internalized homophobia as a personal variable, as this term refers to the process by which same-sex attracted individuals incorporate social representations of gender into their self-image [19]. These representations are based on negative feelings and attitudes toward oneself when the person acknowledges their homosexuality [20]. It is known that higher internalized homophobia, or internalized homonegativity [21], is associated with lower sexual satisfaction [16,22]. Based on published literature, the present study considered attachment dimensions (anxiety and avoidance), sexual functioning, dyadic adjustment, relationship satisfaction, and the components of the Interpersonal Exchange Model of Sexual Satisfaction (IEMSS) [5] as interpersonal variables. Attachment refers to the process of emotional bond experienced by human beings, which stems from the relationship with one’s caregivers [23,24]. It plays a crucial role in an adult’s relationships with peers, causing the tendency to seek and maintain proximity and contact with certain people who provide the person with psychological or physical security [25]. Attachment is composed of two primary dimensions: Abandonment anxiety and avoidance of intimacy [26]. Depending on the degree of anxiety and avoidance, attachment styles are categorized into secure, anxious/ambivalent, and avoidant [27]. The avoidant and anxious attachment styles have been associated with low satisfaction, both sexually and in terms of relationships, in opposite-sex attracted individuals and same-sex attracted individuals [28,29,30]. Sexual functioning is the capacity to adequately respond to sexual stimuli and to participate in pleasurable and pain-free sexual activities [31]. Among the same-sex attracted population, good sexual functioning is associated with high sexual satisfaction [32]. On the other hand, dyadic adjustment reflects the well-being of the couple relationship seen as an entity [33], that is, as a form of general well-being associated with specific components (cohesion, consensus, and satisfaction), related to problem-solving in the relationship [34,35]. Calvillo et al. [32] reported that a better dyadic adjustment is associated with higher sexual satisfaction among same-sex attracted individuals. Relationship satisfaction was defined by Fallis et al., based on the definition of sexual satisfaction by Lawrance and Byers [5], as “an affective response arising from one’s subjective evaluation of the positive and negative dimensions associated with one’s romantic relationship” [36] (p. 822). Several studies have concluded that relationship satisfaction predicts sexual satisfaction in same-sex attracted individuals [16,37,38]. Lastly, in the present study, the IEMSS components were considered as interpersonal variables [5]; therefore, sexual satisfaction is determined by (1) the balance between sexual rewards and costs in the relationship (REW-CST), (2) the comparative level between actual sexual rewards/costs and expected sexual rewards/costs (CL_REW_-CL_CST_), and (3) perceived equality of sexual costs (EQ_CST_) and rewards (EQ_REW_) between the members of the couple. Although not part of the model, the number of sexual rewards and the number of sexual costs were also considered as interpersonal variables.

Sexual minorities have become increasingly visible; as a result, the opportunity to examine different aspects of same-sex couple relationships has increased [39]. Few studies have attempted to explain sexual satisfaction among the LGBT community using multifactorial models [8,12,16]. The study of sexual satisfaction of men and women with a same-sex partner is essential for the development of programs to promote the well-being of a same-sex relationship and to understand gender differences through an explanatory model of Spanish-speaking men and women with a same-sex partner. These gender differences in sexual satisfaction have been shown in the study by Calvillo et al. [32]. However, it was not examined in an explanatory model. Therefore, the aim of the present study was to present an explanatory model of sexual satisfaction in adults with a same-sex partner that (1) integrates personal and interpersonal variables, and (2) evidences gender differences in the associations of personal and interpersonal variables with sexual satisfaction. According to previous research, it was hypothesized that, for the development of the model, the personal variable of internalized homophobia and the interpersonal variables of anxiety, avoidance, and number of sexual costs would negatively associate with sexual satisfaction [22,32,40] at the moment of predicting it, while the interpersonal variables of sexual functioning, dyadic adjustment, relationship satisfaction, the balance between sexual rewards and costs in the relationship, comparison of the actual rewards and costs with those expected, and number of sexual rewards would positively associate when predicting sexual satisfaction [16,17,32].

In addition to the hypothesis, it is worth mentioning that two research questions regarding the aforementioned variables were posed while doing this research:

1. Will personal and interpersonal variables associate (directly and/or indirectly) with sexual satisfaction?
2. Will the explanatory models show gender differences?

## 2. Method

### 2.1. Participants

A sample of 820 Spanish-speaking participants (410 men and 410 women) who had been in a romantic relationship for at least three months with a person of the same sex was obtained by nonprobabilistic convenience sampling. For men, the age range was between 18 and 66 (mean [*M*] = 29.24, standard deviation [*SD*] = 9.84), and for women, between 18 and 58 (mean [*M*] = 29, standard deviation [*SD*] = 8.57). Most participants were Spanish (57.6%); the rest were Mexican (23.7%), Colombian (12%), Chilean (4.3%), Venezuelan (1.1%), Argentinian (0.5%), Dominican (0.2%), Ecuadorian (0.2%), Cuban (0.1%), Honduran (0.1), Paraguayan (0.1), and Peruvian (0.1). Exclusion criteria were: (1) Being a citizen of a non-Spanish-speaking country, (2) having Spanish as a non-native language, (3) being underage, (4) having a sexual orientation that is not towards people of the same sex, (5) discrepancy between biological sex and gender identity in the participant or their partner, (6) do not maintain a romantic relationship for at least three months, and (7) not having sexual activity with the partner at the time of the evaluation. Table 1 presents the participants’ sociodemographic information.

### 2.2. Procedure

Evaluation instruments were administered using both the traditional paper-and-pencil format and the online format. Neither method showed differences in the information collected on sexual behaviors [41]. Participants who completed questionnaires in paper-and-pencil format were approached at public areas and leisure centers, as well as through lesbian, gay, bisexual, and transgender (LGBT) associations. The online version was distributed using virtual platforms (Facebook^®^, Twitter^®^, WhatsApp^®^ groups, and e-mail), using Limesurvey^®^ software (Limesurvey GmbH Hamburg, Germany) and controlling the IP address of the responses; to avoid automated responses, participants were asked to confirm their access to the survey by responding to a security question consisting of a simple randomized arithmetic operation. To distribute the questionnaire via Facebook^®^, a post was published on the Laboratory of Human Sexuality page and promoted to invite users of this social network to participate. Anonymity was guaranteed to all participants, as well as the confidentiality of their data, and their participation was voluntary. Before responding, participants were asked to read and accept an informed consent form, which described the purpose of the study and provided information on data confidentiality and privacy. The study was approved by the University of Granada Human Research Ethics Committee.

### 2.3. Measures

A sociodemographic questionnaire was used to collect information on sex, age, nationality, couple relationship, partner’s sex, length of relationship, cohabitation with partner, sexual relations with current partner, age of first sexual relation and type (anal, oral, or vaginal), and number of sexual partners.

The Kinsey Scale [42] was used to identify sexual orientation based on eight response options, from *Exclusively heterosexual* (1) to *Exclusively homosexual* (7); an eighth option was included to account for asexuality. Only participants who reported being exclusively attracted to people of the same sex were selected.

The Spanish version of the Internalized Homonegativity (IH) scale [43], published by Ortega López [44], which includes four response items, was used to evaluate the participants’ recognition of their own negative opinions about same-sex attraction, that is, heterosexism or internalized homophobia [44]; this scale uses a seven-point Likert-type scale to score the degree of agreement or disagreement with each of the items. Lower scores indicate lower internalized homophobia. Its internal consistency reliability has been reported to be 0.88 [45], and, in the present study, an ordinal alpha of 0.94 was obtained for both men and women. 

The Spanish version of the Experiences in Close Relationship Short-Form (ECR-S) [46], adapted by Calvillo et al. [47], served to measure the dimensions of attachment: Anxiety and avoidance, in the context of couple relationships. Its 12 items are answered on a seven-point Likert scale from *Totally disagree* (1) to *Totally agree* (7). Higher scores indicate higher anxiety or avoidance. The instrument has shown good reliability in same-sex attracted population [47]. In our study sample, the Avoidance subscale obtained an ordinal alpha of 0.79 for men and 0.82 for women, whereas the Anxiety subscale obtained a score of 0.74 for men and 0.76 for women.

The Spanish version of the Massachusetts General Hospital-Sexual Functioning Questionnaire (MGH-SFQ) [48], adapted by Sierra et al. [49], allowed for the evaluation of sexual functioning over the month prior to application through five items focusing on interest, arousal, orgasm, erection (in men), and general sexual satisfaction. Answers are given on a five-point scale with anchors from *Totally decreased* (0) to *Normal* (4). Given that the possible range of scores was different for men and women, the mean score was used instead of the total score. To avoid overlapping between sexual functioning and the measure of sexual satisfaction, item 5 (sexual satisfaction) was excluded from the calculation of the mean. Higher scores indicate good sexual functioning. This questionnaire presents good reliability in the same-sex attracted population [32]. In the present study, ordinal alpha was found to be 0.92 for men and 0.89 for women.

The short Spanish version of the Dyadic Adjustment Scale (DAS) [50], adapted by Santos-Iglesias et al. [51], was employed to evaluate dyadic adjustment in the couple based on three dimensions: Satisfaction, Consensus, and Cohesion. Its 13 items are answered on Likert-type scales including six response options (from *Always disagree to* to *Always agree to*) or five response options (from *Never* to *Every day*), depending on the item. Higher scores indicate better adjustment. It has been shown to be reliable and valid for same-sex attracted population [32], and the present study obtained an ordinal alpha of 0.85 for men and women.

The Spanish version of the Interpersonal Exchange Model of Sexual Satisfaction Questionnaire (IEMSSQ) [52], published by Sánchez-Fuentes et al. [53], and adapted to same-sex attracted population by Calvillo et al. [32], includes four independent measures: The Exchange Questionnaire (EXQ), the Global Measure of Sexual Satisfaction (GMSEX), the Global Measure of Relationship Satisfaction (GMREL), and the Rewards/Costs Checklist (RCC). The IEMSSQ has shown excellent psychometric properties among same-sex attracted respondents [32]. The EXQ is composed of six items, and it evaluates the different components of the IEMSS. These six items use nine-point scales; item 1 focuses on the general degree of sexual reward (REW), with anchors from *Not at all rewarding* (1) to *Extremely rewarding* (9). Item 2 evaluates the actual level of sexual rewards in comparison with the expected level thereof (CL_REW_) with anchors from *Much less rewarding in comparison* (1) to *Much more rewarding in comparison* (9). Item 3 evaluates the perceived level of reward in comparison to one’s couple’s perceived level of reward (EQ_REW_) with anchors from *My rewards are much higher* (1) to *My partner’s rewards are much higher* (9). The three remaining items (4, 5, and 6) are similar to the first three but focused on sexual costs (CST). The total balance between rewards and costs (REW-CST) is the difference between the score of item 1 and the score of item 4. The level of comparison between sexual rewards and costs (CL_REW_-CL_CST_) is calculated by subtracting the score of item 5 from the score of item 2. In both cases, possible scores range from −8 to +8, where higher scores represent more sexual rewards. In the present study, the components of equality of rewards (EQ_REW_) and equality of costs (EQ_REW_) were excluded because they have been shown to have little or no impact on sexually satisfied people [32,54]. The GMSEX evaluates overall satisfaction with one’s sexual relationship using five seven-point bipolar subscales: *Very bad/Very good, Very unpleasant/Very pleasant, Very negative/Very positive, Very unsatisfying/Very satisfying, Very worthless/Very valuable.* The GMREL is identical to the previous measure of satisfaction, but it measures satisfaction with one’s romantic relationship. Finally, the RCC is composed of 58 items, representing different sexual exchanges that can be valued as rewards, costs, both, or neither. The sum total of rewards indicates the number of sexual rewards and the sum total of costs represents the number of sexual costs. In the present study, the GMSEX obtained an ordinal alpha of 0.94 for men and 0.93 for women; concerning the GMREL, an ordinal alpha of 0.95 was obtained for both men and women.

### 2.4. Data Analysis

Firstly, to analyze differences by sex, Student’s *t*-test for independent samples was used for each of the studied variables. Secondly, correlations between the evaluated variables were carried out. In this way it was possible to confirm whether the direction that the associations of the variables had with sexual satisfaction was positive or negative. The nationality variable was included in the aforementioned analysis to determine its relationship with the other variables. Subsequently, all the variables (except for nationality that showed low or no correlations with the rest of the variables) were included in a structural equation model using RStudio software (Version 1.1.447, RStudio Inc., Boston, MA, USA) [55] and the lavaan package (Version 0.6–4, Ghent University, Ghent, Belgium) [56]. The explanatory model was based on the analysis of multiple linear regression models within the framework of the structural equation model (SEM). Because of this, it was possible to request more than one regression analysis simultaneously by using path analysis [57] and, through the decomposition of correlations that is afforded by the path analysis, the interpretation of the relationships was enhanced as well as the pattern of the effects of one variable on another [57]. In this way, the estimates of the multiple regression parameters and the types of results reported were determined [58]. This put on evidence the direct, indirect, and total effect of the variables [57]. In some studies [59,60,61], SEM analysis has been used to evaluate predictions and, as a result, the coefficient of determination (*R*^2^) has been shown without the fit indexes that are commonly evidenced. Therefore, for the development of explanatory or predictive models, the variance portion can be used as a unique criterion to show the explanatory, or predictive, capacity in models that are developed with SEM. Given the noncompliance of the multivariate normality, and designed for ordinal data [62], the analysis included Maximum Likelihood estimation with robust standard errors (MLM), which uses the Satorra-Bentler scaled test statistic [63]. In all the analyses, *p* values below 0.05 indicate statistical significance.

## 3. Results

### 3.1. Descriptive Statistics and Differences by Sex

Significant sex-based differences were found in nine of the 11 analyzed variables, all of them interpersonal: Anxiety, avoidance, sexual functioning, dyadic adjustment, sexual satisfaction, relationship satisfaction, REW-CST, CL_REW_-CL_CST_, and number of sexual costs. No significant differences were found between men and women in regard to internalized homophobia (personal variable) and number of sexual rewards (interpersonal variable). Men reported better sexual performance than women, whereas women reported more dyadic adjustment, sexual satisfaction, and relationship satisfaction, as well as more rewards than sexual costs (REW-CST) and a higher relative level of rewards than cost (CL_REW_-CL_CST_); compared to men, they reported lower anxiety, avoidance, and fewer sexual costs than men. However, both men and women reported good sexual and relationship satisfaction, as well as good dyadic adjustment and sexual functioning. Additionally, all participants presented low anxiety, avoidance, and internalized homophobia (Table 2).

### 3.2. Bivariate Correlations

Regarding correlations, negative associations were found between sexual satisfaction and internalized homophobia, anxiety, avoidance, and number of sexual costs. While positive associations were found in sexual satisfaction with sexual functioning, dyadic adjustment, relationship satisfaction, REW-CST, CL_REW_-CL_CST_, and number of sexual rewards. All the correlations of the variables showed significance when they were associated with sexual satisfaction, except for the correlation of the nationality variable. Furthermore, regarding the strength of the associations in the different variables that were associated with sexual satisfaction in men and women with a same-sex partner, gender differences were evident. The nationality variable presented low or non-existent correlations with the other analyzed variables (Table 3).

### 3.3. Testing the Models

Given the sex-based differences in virtually all evaluated variables, independent models were created for men and women. Sexual satisfaction was the observable dependent variable in both models, whereas the rest of the personal (internalized homophobia) and interpersonal (anxiety, avoidance, sexual functioning, dyadic adjustment, relationship satisfaction, IEMSS components (REW-CST and CL_REW_-CL_CST_), number of sexual rewards, and number of sexual costs) observable variables were the independent ones.

**Figure 1:**
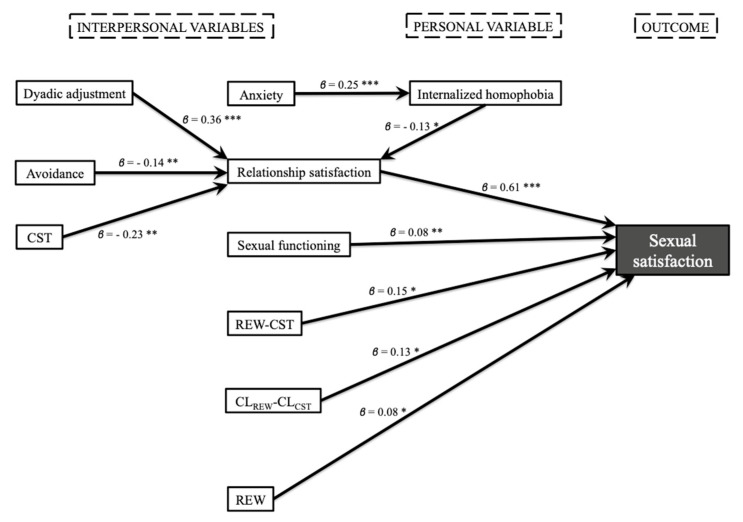
Explanatory model of sexual satisfaction in men with a same-sex partner (*n* = 410, * *p* < 0.05, ** *p* < 0.01, *** *p* < 0.001).

In the men’s model, sexual functioning (*β* = 0.08), relationship satisfaction (*β* = 0.61), REW-CST (*β* = 0.15), CL_REW_-CL_CST_ (*β* = 0.13), and number of sexual rewards (*β* = 0.08) were directly and positively associated with sexual satisfaction. Additionally, the model included variables indirectly related to sexual satisfaction, as well as mediating variable. It was thus made apparent that relationship satisfaction acts as a mediating variable in the association between sexual satisfaction and internalized homophobia (*β* = −0.13), avoidance (*β* = −0.14), dyadic adjustment (*β* = 0.36), and number of sexual costs (*β* = −0.23); except for number of sexual costs, all associations were positive. In turn, internalized homophobia acted as a mediating variable between anxiety (*β* = 0.25) and relationship satisfaction (Figure 1). Finally, the model explained 67.8% of the variance in sexual satisfaction.

**Figure 2:**
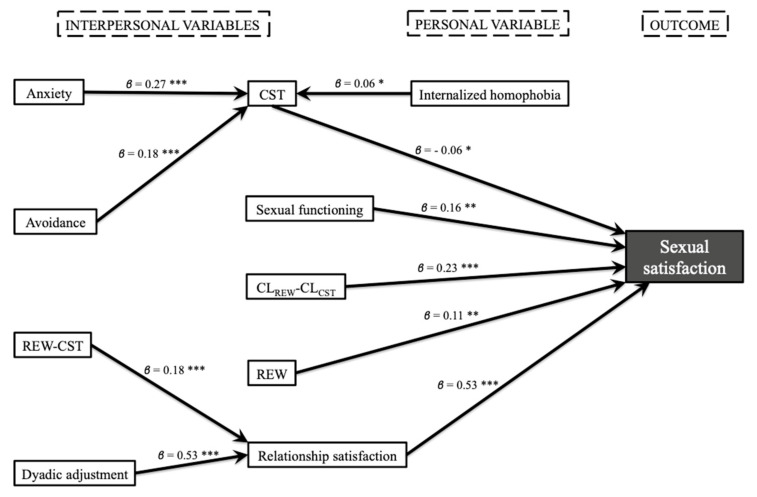
Explanatory model of sexual satisfaction in women with a same-sex partner (*n* = 410, * *p* < 0.05, ** *p* < 0.01, *** *p* < 0.001).

In the women’s model, sexual satisfaction was directly determined by sexual functioning (*β* = 0.16), relationship satisfaction (*β* = 0.53), CL_REW_-CL_CST_ (*β* = 0.23), number of sexual rewards (*β* = 0.11), and number of sexual costs (*β* = −0.06); in all cases, the association was positive, except for number of sexual costs. Number of sexual costs was found to act as a mediating variable between sexual satisfaction and internalized homophobia (*β* = 0.06), anxiety (*β* = 0.27), and avoidance (*β* = 0.18), all of them positively associated with CST. Relationship satisfaction was positively affected by dyadic adjustment (*β* = 0.53) and REW-CST (*β* = 0.18), acting as a mediating variable between these two variables and sexual satisfaction (Figure 2). The women’s model explained 58.8% of the variance in sexual satisfaction.

## 4. Discussion

The purpose of the present study was to create an explanatory model of sexual satisfaction in people with a same-sex partner using the Ecological theory [13] as a reference, which has been carried out in previous studies focused on heterosexuals [17]. Given that they are the most important variables to explain sexual satisfaction [3,4], the model included personal (i.e., internalized homophobia) and interpersonal (i.e., anxiety, avoidance, sexual functioning, dyadic adjustment, relationship satisfaction, and IEMSS components) variables. In general, the study participants reported high levels of sexual satisfaction, relationship satisfaction, good dyadic adjustment, adequate sexual functioning, low levels of avoidance and anxiety (secure attachment style), and little internalized homophobia. Nevertheless, except for internalized homophobia, differences between men and women were found in all the aforementioned variables.

The low levels of internalized homophobia (the only personal variable included in the study) reported by the study participants and the lack of differences by sex could be due to a secure attachment style [64] or perceived good quality in their relationships [65], which would act as protective factors against the negative internalization of prejudices toward same-sex attraction. The low level of anxiety and avoidance (i.e., secure attachment), high degree of relationship satisfaction, and low level of internal homophobia found in the study sample support this hypothesis.

In terms of interpersonal variables, in general, both men and women obtained low anxiety and avoidance scores, which suggests secure attachment. In the case of women, our results are comparable to those reported by Guzmán-González et al. [66]. According to Attachment theory in adults [23,27], secure attachment is associated with greater confidence, closeness, and relationship satisfaction [27,47]. Therefore, one would logically infer that greater relationship satisfaction is associated with less anxiety and avoidance, as indicated by the results. However, men reported higher levels of anxiety and avoidance than women, which is consistent with results reported by Guzmán-González et al. [66], Gabbay and Lafontaine [67], Mohr et al. [68], and Ridge and Feeney [69]. These differences between men and women could be due to difficulties in same-sex relationships between men, such as intimacy issues in the relationship associated with restrictive norms of the masculine gender [68,70], emotional disconnection [71], or competition between both partners [66]. All of this could result in patterns characteristic of anxious attachment, such as insecurity, physical and psychological tolerance toward insults, communicating exclusively positive feelings and avoiding negative ones, and need for attention [72], or in behaviors typical of avoidant attachment styles, such as physical and emotional independence and distance and difficulty in distinguishing emotions [72]. 

On the other hand, men obtained better sexual functioning scores than women, as has been the case in studies conducted with heterosexual populations [49,73,74,75]. However, women obtained better sexual satisfaction scores than men. This reinforces the fact that a woman’s sexual satisfaction, regardless of sexual orientation, is related not only with her physical sexual response, but also with other factors that may have more to do with intimacy, affection, and emotional closeness [3,17,74,76,77]. The fact that same-sex attracted individuals report, in general, high sexual and relationship satisfaction has already been reported by Calvillo et al. [32]. In the same study, the authors also report that, in comparison with men with a same-sex partner, women with a same-sex partner feel more sexually satisfied (as indicated by the overall measure and IEMSSQ components) and satisfied with their relationship, but no sex-based differences were found in heterosexuals [54].

Concerning the sexual satisfaction explanatory models, few variables were found to be directly associated with sexual satisfaction. For men and women, these variables were sexual functioning, relationship satisfaction, comparison of sexual rewards and costs, and number of sexual rewards; specifically for men, sexual satisfaction was related to the balance between rewards/costs, and for women, to the number of sexual costs (research question 1). As hypothesized, all the variables were positively associated with sexual satisfaction, except for number of sexual costs, whose association was negative. These associations are consistent with previous research focused on same-sex attracted individuals [9,16,32,78].

In both models, relationship satisfaction was found to have not only the highest weight in explaining sexual satisfaction (*β =* 0.61 in men and *β =* 0.53 in women) but also a mediating role between other variables and sexual satisfaction (research question 1). This is more relevant in the case of men because all the variables not directly associated with sexual satisfaction were instead associated with relationship satisfaction, that is, internalized homophobia, avoidance, dyadic adjustment, and number of sexual costs (research questions 1 and 2); in the case of women, relationship satisfaction was associated with dyadic adjustment and balance between sexual rewards and costs (research questions 1 and 2). As could be expected, the variable of dyadic adjustment (*β =* 0.36 in men and *β* = 0.53 in women) had the strongest influence on relationship satisfaction. Dyadic adjustment and relationship satisfaction refer to the quality of the romantic relationship; therefore, the close positive relationship between the two variables is logical and has been confirmed in other studies on same-sex attracted population [47,79]. The fact that the association between these two variables was higher in women than in men could be due to the characteristics of the couple relationship in women with a same-sex partner; for example, women with a same-sex partner are characterized by extreme emotional closeness between partners [80], a high degree of intimacy [81], strong dyadic cohesion [82], and better communication patterns concerning sexual life [83]. Additionally, women are more likely to “open up” or communicate more intimately and emotionally than men [84,85]. Therefore, communication could be expected to be better among women in same-sex partner relationships than among men with same-sex partner relationships, which would strengthen emotional intimacy in woman-woman relationships and this should lead to a better dyadic adjustment. 

Among women, in addition to relationship satisfaction, the number of sexual costs acts as a mediating variable between internalized homophobia, anxiety, avoidance, and sexual satisfaction (research questions 1 and 2). The fact that this mediating factor appears in the women’s model but not in the case of men could be due to several reasons. Concerning internalized homophobia, Cohen et al. [86] identified feelings of guilt about having desires towards people of the same sex as a possible cost for same-sex attracted individuals. Guilt produced by homophobia appears to be an important cost in the sexual relationships of the women in the present study. Regarding anxiety and avoidance, Stephenson and Meston [87] found a relationship between anxiety and cost in heterosexual women as well. A possible explanation is that negative relational-affective characteristics of anxiety in the context of sexual relationships, such as the desire to obtain reassurance [88] or affection [89] through sex with the partner or, as has been shown for women, engaging in affectionless sexual activity [90], could exacerbate people’s concerns about sex with their partner, especially in women. As for avoidance, it refers to discomfort about sexual intimacy with a partner [91], which could result in forced sexual activity [92]; in women, such discomfort would interfere with sexual satisfaction. Therefore, women appear to bear higher sexual costs due to anxiety and avoidance than men. In all cases, the direction of the associations was as hypothesized. Calvillo et al. [3] have reported that internalized homophobia and anxious or avoidant attachment are negatively associated with sexual satisfaction; therefore, in women with a same-sex partner, these variables could increase costs and negatively affect their sexual satisfaction.

In men, anxiety acts indirectly through internalized homophobia and relationship satisfaction (research questions 1 and 2); the direction of these associations is also logical and as expected according to the hypothesis presented. The fact that anxiety has been associated with internalized homophobia in men but not in women could be due to several reasons. Attachment theory in adults [27] states that the anxious attachment style is based on a negative model of oneself and positive models about others [24], the negative impact of homophobia is known to be higher on men than on women [93,94], and lesbian women tend to be more resilient to homophobic experiences than gay men due to their increased social support [93]; this would suggest that, as an attachment style, anxiety decreases resources for same-sex attracted men to cope with homophobia, resulting in a more intense experience of internalized homophobia compared to women. The positive relationship between anxiety and internalized homophobia confirms the fact that individuals who do not accept their sexual orientation tend to display avoidance or anxiety (insecure attachment styles) [95], whereas the presence of secure attachment has been shown to increase self-esteem and resilience, thus decreasing internalized homophobia [96]. The low weight of internalized homophobia in both the men’s and the women’s models suggests that, for same-sex attracted individuals with high levels of sexual satisfaction and relationship satisfaction, as well as low levels of anxiety and avoidance, internalized homophobia has little relevance on sexual satisfaction.

In both models, relational and emotional variables (relationship satisfaction and dyadic adjustment) were found to explain most of sexual satisfaction, which makes them relevant variables when considering sexual satisfaction in the context of same-sex relationships. According to previous studies on same-sex attracted [9,16,97] and opposite-sex attracted [17,98,99] populations, the fact that sexual satisfaction can be predicted on the basis of relationship satisfaction suggests that both constructs are closely related. Relationship satisfaction represents a reward for the couple [5]; therefore, if this reward is high, sexual satisfaction is also expected to be high, as has been shown in other studies [100,101]. Nevertheless, variables not analyzed in this study, such as sexual compatibility [98] or sexual and nonsexual communication [102], could also be mediating variables between these two types of satisfaction.

## 5. Limitations

The present study had some limitations. In the first place, this investigation had a nonprobabilistic convenience sampling; therefore, the generalizability of the sample was limited and not representative. Secondly, due to the design of this research, the results did not establish causality effects since sexual satisfaction was assessed at a specific time. Thirdly, although people reported having a specific sexuality at the time of participating in this study, it is important to note that sexuality and relationship are fluid and dynamic. In the fourth place, only one personal variable was used; therefore, the study would need to be replicated using more personal variables (for instance, a measure evaluating outness, the sense of belonging to the LGBT community, or a psychopathological dimension such as depression or anxiety). Fifth, all the associations showed unidirectional relationships, so it is suggested in future studies to incorporate reciprocal causal relationships in the associations that require it. Furthermore, sexual satisfaction was studied independently of the dyad formed by the couple; therefore, future research should focus on the sexual satisfaction of same-sex couples using actor-partner interdependence models (AIPM) to showcase the influence of one partner’s variables on the other’s [54,103]. In addition, sexual satisfaction is known to decrease with age [4]; the proposed explanatory models of sexual satisfaction in people with same-sex couples should be used in different age groups, as has been done with other dimensions of sexuality, such as sexual desire [104] or the subjective experience of orgasm [105]. Finally, the model was developed exclusively for cisgender people; therefore, studies examining an explanatory model of sexual satisfaction among different groups within the LGBT community (for example, transgender or intersex people) would be advisable, as well as studies focusing on serodiscordant couples including variables associated with HIV. 

## 6. Conclusions

This study proposed explanatory models of sexual satisfaction for men and women with a same-sex romantic partner. The models included personal and interpersonal variables, in which the IEMSS components [5] and variables associated with relational aspects play an essential role. These explanatory models could be used to support future research analyzing sexual satisfaction in same-sex attracted populations. They can also be used for the development of effective therapies to potentiate variables positively associated with sexual satisfaction and inhibit those that associate negatively with the aim of improving sexual health, the quality of sexual relationships between men and women with a same-sex partner, and dyadic sexual well-being. The identified mediating variables are also relevant for clinicians, since intervention in a variable often promotes changes in other variables (e.g., an intervention focused on dyadic adjustment would have effects not only on relationship satisfaction but also on sexual satisfaction).

In sum, sexual satisfaction is regulated by both personal and interpersonal factors, with the relational aspects having the highest weight. These results are in line with studies showing that dyadic processes are a fundamental characteristic in the sexual satisfaction of people in a romantic relationship [6,106].

## Figures and Tables

**Table 1:** Sociodemographic characteristics of the participants (*n* = 820).

Variables	Men (*n* = 410)		Women (*n* = 410)		
	Rank	*M (SD)*	Rank	*M (SD)*	*t/* *χ^2^*
Age (years)	18–66	29.24 (9.84)	18–58	29 (8.57)	0.36
	***M (SD)***		***M (SD)***		
First sexual relation (years)	16.52 (3.82)		17.55 (3.12)		−4.21 ***
Duration of the relationship with current partner (months)	47.70 (56.75)		46.90 (50.32)		0.21
	***M_e_***	***M (SD)***	***M_e_***	***M (SD)***	
Number of sexual partners	11.50	32.18 (66.03)	5	7.51 (10.73)	7.46 ***
**Nationality**	***n* (%)**		***n* (%)**		
		
Spanish	225 (54.90)		247 (60.20)		2.41
Other Hispanic countries	185 (45.10)		163 (39.80)		
**Education level**					
Basic	6 (1.50)		10 (2.4)		1.32
Intermediate	113 (27.60)		105 (25.60)		
Higher	291 (71)		295 (72)		
**Cohabit with current partner**					
Yes	232 (56.60)		231 (56.30)		0.005
No	178 (43.40)		179 (43.70)		

Note. M: Mean; SD: standard deviation; M_e_: median; *** *p* < 0.001.

**Table 2:** Comparisons by sex of the evaluated variables.

Variables		Men (*n* = 410)	Women (*n* = 410)	
	Rank	*M (SD)*	*M (SD)*	*p*
Internalized homophobia	4–28	6.50 (4.64)	6.18 (4.33)	0.309
Anxiety	6–42	20.88 (8.09)	18.37 (7.63)	<0.001
Avoidance	6–42	13.50 (6.27)	10.84 (5.07)	<0.001
Sexual functioning	0–20 (Men) 0–16 (Women)	13.75 (3.27)	10.20 (2.46)	<0.001
Dyadic adjustment	12–75	61.01 (7.96)	63.28 (6.81)	<0.001
Sexual satisfaction	5–35	29.99 (6.35)	32.10 (4.30)	<0.001
Relationship satisfaction	5–35	30.48 (6.35)	32.11 (4.68)	<0.001
REW-CST	from −8 to +8	3.80 (3.53)	5.07 (3.06)	<0.001
CL_REW_-CL_CST_	from −8 to + 8	3.10 (3.44)	4.60 (3.10)	<0.001
REW	0–58	43.63 (7.92)	44.55 (6.52)	0.070
CST	0–58	15.58 (9.07)	13.02 (7.57)	<0.001

Note. M: Mean; SD: standard deviation; REW-CST: balance between sexual rewards and costs; CL_REW_-CL_CST_: comparative level of sexual reward and costs; REW: number of sexual rewards; CST: number of sexual costs.

**Table 3:** Correlations between sexual satisfaction with personal and interpersonal variables and with the nationality variable in men and women with a same-sex partner.

Variables	Outcome	Personal Variable	Interpersonal Variables									
	1	2	3	4	5	6	7	8	9	10	11	12
1. Sexual satisfaction	1	−0.18 **	−0.21 **	−0.36 **	0.40 **	0.46 **	0.77 **	0.59 **	0.54 **	0.46 **	−0.48 **	−0.05
2. Internalized homophobia	−0.22 **	1	0.25 **	0.28 **	−0.13 **	−0.24 **	−0.27 **	−0.14 **	−0.10 *	−0.08	0.07	0.20 **
3. Anxiety	−0.22 **	0.15 **	1	0.30 **	−0.11 *	−0.29 **	−0.27 **	−0.29 **	−0.24	−0.18 **	0.19 **	0.09
4. Avoidance	−0.33 **	0.24 **	0.38 **	1	−0.30 **	−0.54 **	−0.43 **	−0.39 **	−0.33 **	−0.31 **	0.31 **	0.18 **
5. Sexual functioning	0.42 **	−0.04	−0.15 **	−0.17 **	1	0.38 **	0.30 **	0.40 **	0.34 **	0.36 **	−0.42 **	−0.11 *
6. Dyadic adjustment	0.44 **	−0.26 **	−0.37 **	−0.51 **	0.24 **	1	0.55 **	0.46 **	0.41 **	0.43 **	−0.42 **	−0.05
7. Relationship satisfaction	0.68 **	−0.22 **	−0.27 **	−0.37 **	0.24 **	0.59 **	1	0.46 **	0.40 **	0.37 **	−0.43 **	−0.08
8. REW-CST	0.49 **	−0.09	−0.27 **	−0.33 **	0.45 **	0.37 **	0.38 **	1	0.80 **	0.48 **	−0.54 **	−0.07
9. CL_REW_-CL_CST_	0.54 **	−0.11 *	−0.30 **	−0.30 **	0.40 **	0.39 **	0.39 **	0.72 **	1	0.40 **	−0.46 **	−0.05
10. REW	0.39 **	−0.08	−0.25 **	−0.17 **	0.26 **	0.22 **	0.27 **	0.35 **	0.30 **	1	−0.62 **	−0.05
11. CST	−0.44 **	0.15 **	0.35 **	0.30 **	−0.37 **	−0.34 **	−0.33 **	−0.47 **	−0.39 **	−0.62 **	1	0.09
12. Nationality	0	0.14 **	0.05	0.16 **	0.09	−0.07	−0.08	−0.03	0	−0.02	0.16 **	1

Note. REW-CST: balance between sexual rewards and costs; CL_REW_-CL_CST_: comparative level of sexual rewards and costs; REW: number of sexual rewards; CST: number of sexual costs. Above the diagonal are the correlations in men and below the diagonal are correlations in women; * *p* < 0.05; ** *p* < 0.01.
